# Heart Failure With Preserved Ejection Fraction: A Review of Cardiac and Noncardiac Pathophysiology

**DOI:** 10.3389/fphys.2019.00638

**Published:** 2019-05-29

**Authors:** Andreas B. Gevaert, Jente R. A. Boen, Vincent F. Segers, Emeline M. Van Craenenbroeck

**Affiliations:** ^1^ Research Group Cardiovascular Diseases, GENCOR Department, University of Antwerp, Antwerp, Belgium; ^2^ Department of Cardiology, Antwerp University Hospital (UZA), Edegem, Belgium; ^3^ Laboratory of Physiopharmacology, University of Antwerp, Antwerp, Belgium

**Keywords:** heart failure, pathophysiology, comorbidities, endothelium, microRNA, iron deficiency, exercise intolerance

## Abstract

Heart failure with preserved ejection fraction (HFpEF) is one of the largest unmet clinical needs in 21st-century cardiology. It is a complex disorder resulting from the influence of several comorbidities on the endothelium. A derangement in nitric oxide bioavailability leads to an intricate web of physiological abnormalities in the heart, blood vessels, and other organs. In this review, we examine the contribution of cardiac and noncardiac factors to the development of HFpEF. We zoom in on recent insights on the role of comorbidities and microRNAs in HFpEF. Finally, we address the potential of exercise training, which is currently the only available therapy to improve aerobic capacity and quality of life in HFpEF patients. Unraveling the underlying mechanisms responsible for this improvement could lead to new biomarkers and therapeutic targets for HFpEF.

## Introduction

Heart failure (HF) is a complex clinical syndrome that results from a structural or functional impairment of contraction or filling of the heart. It is a very common condition: 1–3% of the adult population has HF, and the prevalence rises with age (Dunlay et al., 2017). The chief symptoms of HF are exercise intolerance and dyspnea on exertion. Fatigue, peripheral edema, orthopnea, paroxysmal nocturnal dyspnea, loss of appetite, and nycturia are other possible signs and symptoms.

Current guidelines divide HF patients according to left ventricular (LV) ejection fraction (defined as LV stroke volume over LV end-diastolic volume) (Yancy et al., 2013; Ponikowski et al., 2016). Signs and symptoms are equal in those with reduced and preserved ejection fraction, but there are differences in pathophysiology and treatment. Patients with HF and reduced ejection fraction (HFrEF) have a prominent LV contraction problem. Fatigue and exercise intolerance are directly caused by the reduced systolic function as the low cardiac output is insufficient to meet the body’s demands. Congestion is also directly caused by the reduced contractility: blood accumulating in the LV causes end-diastolic pressure to rise. This higher pressure is transferred to the pulmonary, portal, and peripheral circulation, where extravasation of fluid causes lung, splanchnic, and peripheral edema.

In patients with HF and preserved ejection fraction (HFpEF), LV ejection fraction is normal, although contractile dysfunction is often present and only detected with advanced imaging techniques. End-diastolic pressure elevation and congestion are as severe as in HFrEF (Van Aelst et al., 2018). In HFpEF, the rise in end-diastolic pressure is caused by a complex interplay between diastolic dysfunction, subtle systolic dysfunction, atrial and LV stiffness, and reduced arterial compliance. The LV, the left atrium, the aorta, and peripheral arteries all participate (Borlaug, 2014). This interaction is explained in detail below.

HF symptoms are more subtle in HFpEF than in HFrEF and often only present on exertion, a fact that often delays diagnosis. Prognosis is, however, equally grim as in HFrEF: 5-year mortality is around 75%, which is worse than most cancers (Shah et al., 2017). Neurohumoral drugs, device therapy, and cardiac rehabilitation have improved survival rates in HFrEF, but not a single treatment has been able to consistently improve prognosis in HFpEF. Guidelines currently advise to treat symptoms with diuretics, and to control comorbidities such as hypertension and diabetes tightly (Yancy et al., 2013; Ponikowski et al., 2016).

Age-standardized incidence rates of HF are more or less stable. Still, due to aging of the population and increasing presence of cardiovascular risk factors, the prevalence of HFpEF will steadily increase in the coming decades (Dunlay et al., 2017). These epidemic proportions, together with the lack of treatment, make HFpEF one of the greatest unmet needs in 21st-century cardiology.

In this review, we give an overview of HFpEF pathophysiology. First, we outline the underlying causes of exercise intolerance in HFpEF. Three players in this process are highlighted: the endothelium, comorbidities, and microRNAs. We critically review existing evidence and address gaps in our current knowledge for each section. Finally, we anticipate on the potential effects of exercise training in HFpEF.

## Cardiovascular Structural and Functional Alterations in Heart Failure With Preserved Ejection Fraction

**Figure 1:**
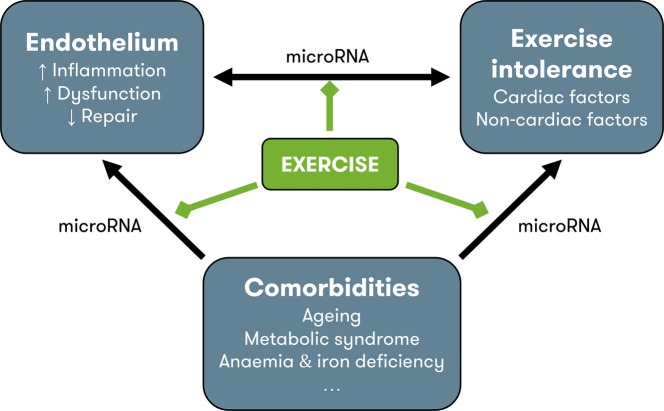
Pathophysiology of HFpEF and possible contribution of exercise training. Endothelial dysfunction contributes to exercise intolerance through noncardiac disturbances (see Figure 2) and modulation of cardiac function, through the nitric oxide – soluble guanylyl cyclase pathway (see Figure 3). Comorbidities also contribute to exercise intolerance, either directly, or indirectly by inducing vascular inflammation and endothelial dysfunction and by impeding endothelial repair. MicroRNAs could play a regulatory role at each level of interaction. In other cardiovascular disorders, exercise is known to reduce inflammation, improve endothelial function, and increase levels of circulating endothelium-repairing cells. Possibly, the improvement in exercise tolerance with exercise training in heart failure with preserved ejection fraction is due to the beneficial effects on the endothelium. Other possible beneficial effects of exercise training include direct improvement of cardiac factors (chronotropic incompetence, diastolic function), noncardiac factors (arterial stiffness, muscle function), and comorbidities (metabolic syndrome), although improvement of endothelial function could be the physiologic base of all these effects. HFpEF = heart failure with preserved ejection fraction.

**Figure 2:**
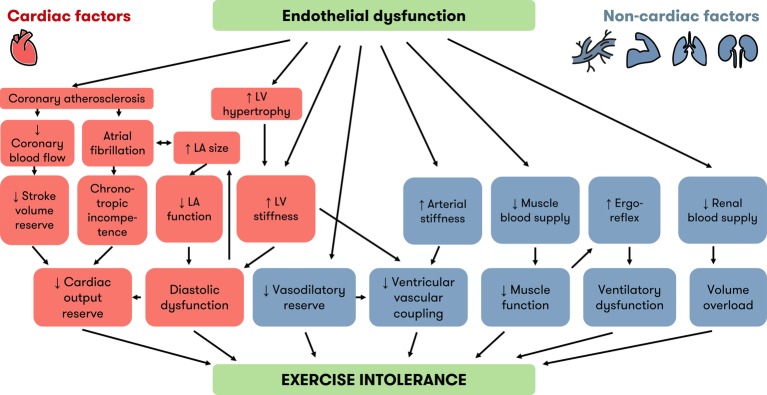
Cardiac and noncardiac factors linking endothelial dysfunction and exercise intolerance in HFpEF. Besides diastolic dysfunction, which is well known, recent evidence implicates other cardiac (orange) and noncardiac (blue) factors in the development of exercise intolerance in HFpEF. Endothelial dysfunction is an underlying mechanism of many factors associated with exercise intolerance. The “inflammatory microvascular dysfunction” hypothesis puts endothelial dysfunction at the root of LV hypertrophy and LV stiffness. Endothelial dysfunction is also a precursor of atherosclerosis and contributes to many noncardiac factors implicated in exercise intolerance. HFpEF = heart failure with preserved ejection fraction, LA = left atrium, LV = left ventricle.

Several pathophysiologic mechanisms lead to an increased LV end-diastolic pressure in HFpEF, and thus cause HF symptoms (Figure 1). Most patients exhibit several pathophysiological abnormalities in a complex interplay, although one mechanism may be more prominent in a single patient (Borlaug et al., 2010). This includes cardiac (diastolic dysfunction, reduced cardiac output reserve, atrial fibrillation, coronary artery disease), and noncardiac elements (reduced vasodilation, arterial stiffness, ventilatory dysfunction, skeletal myopathy, activation of the autonomic nervous system, and renal dysfunction) (Figure 2). A detailed explanation of all these factors falls outside the scope of this review, but we describe the most important contributors to exercise intolerance in HFpEF below.

### Diastolic Dysfunction and Ventricular Stiffness

All patients with HFpEF exhibit some degree of diastolic dysfunction, and diastolic dysfunction is considered a precursor of symptomatic HFpEF. The diastole or filling phase of the cardiac cycle can be divided in two parts. First, the LV changes from a contracted to a fully relaxed state. This is called the “active relaxation” phase, because cardiomyocyte relaxation is an energy-consuming process. The second phase of diastole is called “passive stiffness.” No energy is consumed, but the LV passively stretches under the influence of further filling (Segers and De Keulenaer, 2013). A landmark invasive hemodynamic study by Zile et al. showed that HFpEF patients had both impaired active relaxation and increased passive stiffness (Zile et al., 2004). These findings were later confirmed in a community-based study comparing HFpEF patients to age- and comorbidity-matched controls (Lam et al., 2007). The matching to controls is important because mild diastolic dysfunction is often present in elderly adults without HF symptoms.

In the absence of mitral valve disease, left atrial pressure reflects LV end-diastolic pressure. Long-standing left atrial hypertension leads to left atrial dilation, which is used as a marker of long-term diastolic dysfunction. HFpEF patients are characterized by an increased left atrial volume at rest and reduced left atrial filling on exertion (Reddy et al., 2017).

### Reduced Cardiac Output Reserve

Despite a normal ejection fraction, HFpEF patients often have subtle signs of systolic dysfunction. Global longitudinal strain, a very sensitive parameter of LV contraction, is reduced at rest in HFpEF patients (Kraigher-Krainer et al., 2014). More dramatic systolic abnormalities become evident on exertion: load-independent parameters of LV contractility are reduced by 65% at peak exercise in HFpEF patients (Borlaug et al., 2010). Also, the exercise-induced increase in heart rate is lower than in controls. The latter is called chronotropic incompetence and can be influenced by the concomitant use of beta blockers. Reduced contractile reserve and chronotropic incompetence combine in a reduced cardiac output reserve, which contributes to exercise intolerance in HFpEF (Borlaug, 2014).

### Arterial Stiffness, Reduced Vasodilator Reserve, and Ventricular-Arterial Coupling

Apart from the cardiac abnormalities mentioned above, the blood vessels also play a vital role in HFpEF pathophysiology. Increased stiffness is not only seen in the LV, but also in large arteries such as the aorta. Invasive measurement of arterial waveforms shows reduced arterial compliance and higher arterial elastance at rest in HFpEF patients, independent of blood pressure (Reddy et al., 2017). Arterial stiffening correlates well with end-diastolic pressure and cardiac output reserve. On exertion, increased pressure wave reflections and exaggeration of the abnormal compliance and elastance are seen.

An important function of normal blood vessels is to vasodilate on exertion, to meet the increased oxygen (O_2_) demands of skeletal muscles. This reactive vasodilation is regulated by shear stress on the endothelial cells and is impaired in almost half of HFpEF patients (Borlaug et al., 2010). Patients with impaired vasodilation have a worse prognosis compared to patients with a normal vasodilatory response (Akiyama et al., 2012).

In the normal heart, cardiac and vascular reserves together maintain an efficient ventricular-arterial coupling during exercise. In HFpEF, however, contractile and vascular reserve impairments lead to an abnormal ventricular-arterial coupling. The arterial elastance-end-systolic elastance ratio is reduced less and cardiac output is increased less in HFpEF patients (Borlaug et al., 2010). The most obvious effect of this mismatch is a lower increase in blood pressure on exertion in HFpEF patients.

## Cellular Alterations Underlying Structural and Functional Changes in Heart Failure With Preserved Ejection Fraction

The complex interplay of structural and functional changes outlined above can be explained by pathologic alterations in different cardiac and noncardiac cells, including cardiomyocytes, fibroblasts, and endothelial cells. Also, cross talk between these cell types is thoroughly altered in HFpEF, as is reviewed elsewhere (Segers et al., 2018). Here we focus on the endothelium, as is seems to play a central role in HFpEF pathophysiology. Building on early work by Brutsaert in the 1980’s (Brutsaert et al., 1988), Paulus and Tschöpe formulated the “inflammatory coronary microvascular dysfunction” hypothesis. This states that cardiac stiffness and LV remodeling in HFpEF are not caused by intrinsic cardiac changes, as in HFrEF, but develop because of changes in the cardiac endothelium (Paulus and Tschöpe, 2013).

### Endothelial Cells

#### Normal Endothelial Function

The endothelium is the innermost layer of the blood vessels, present from the smallest capillary to the aorta. More than just a protective layer between the blood and extravascular tissues, endothelial cells are dynamic, highly interactive cells that regulate vascular function and homeostasis (Segers et al., 2018). The healthy endothelium prevents platelet aggregation and leukocyte adhesion, inhibits smooth muscle proliferation, and regulates vascular tone through release of vasoactive substances. These processes are largely mediated by nitric oxide (NO), the main endothelial effector molecule.

NO has the unique property of being a gaseous signaling molecule, thus being able to diffuse quickly into neighboring cells. This property is exploited in the mechanism of endothelium-dependent vasorelaxation: increased blood flow and the accompanying shear stress induce increased NO production and release from endothelial cells, which diffuses into vascular smooth muscle cells. There, NO activates soluble guanylyl cyclase (sGC) and its second messenger cyclic guanosine monophosphate (cGMP), producing relaxation of the vascular smooth muscles and widening of the blood vessel (Segers et al., 2018).

#### Endothelial Dysfunction

In the setting of cardiovascular risk factors (aging, hypertension, diabetes, obesity, dyslipidemia, and smoking), endothelial homeostasis is disturbed (Chistiakov et al., 2015). These risk factors all increase oxidative stress at the level of the endothelium. Reactive oxygen species (ROS) directly react with NO, forming peroxynitrite (ONOO^−^) and reducing NO bioavailability. ROS also uncouple endothelial NO synthase, which starts to produce highly reactive superoxide ($O_{2}^{-}$) instead of NO, further increasing oxidative stress. This vicious circle leads to a vasoconstricting, pro-inflammatory, and pro-thrombotic state called “endothelial dysfunction” (Chistiakov et al., 2015; Gevaert et al., 2017a).

Endothelial dysfunction is considered the first step in the atherosclerotic process and a precursor to overt cardiovascular disease (Leucker and Jones, 2014). Clinically, endothelium-dependent vasodilation measured by ultrasound (flow-mediated dilation, FMD) or peripheral arterial tonometry (PAT) predicts cardiovascular events (Bonetti et al., 2004; Ras et al., 2013).

#### Endothelial Repair

Normal turnover of endothelial cells is slow, lasting at least 47 days (Hobson and Denekamp, 1984). This turnover is not only dependent on proliferation of existing endothelial cells. In 1997, Asahara et al. discovered that a subgroup of CD34^+^ cells form tube-like structures and express endothelial markers *in vitro* (Asahara et al., 1997). These endothelial progenitor cells (EPCs) are derived from the bone marrow and circulate in low numbers in the bloodstream under normal circumstances. They can be recruited to ischemic or damaged endothelium, where they participate in endothelial repair (Van Craenenbroeck and Conraads, 2010). Through growth factor secretion and other paracrine signaling, EPCs promote proliferation of resident endothelial cells and neovascularization. Some authors even suggest that EPCs are able to differentiate into endothelial cells and integrate into the endothelial cell layer (Hristov et al., 2003).

*In vitro*, EPCs require presence of CD3^+^ CD31^+^ T lymphocytes for optimal growth (Hur et al., 2007). These “angiogenic” T cells (TAs) or circulating angiogenic cells are also derived from the bone marrow. TAs secrete high amounts of pro-angiogenic factors (vascular endothelial growth factor, IL-8) and are thought to participate in endothelial repair through paracrine control of EPCs via CD184 (Walter et al., 2005). We have recently shown that numbers of circulating EPCs and TAs are reduced in HFpEF patients, indicating a deficient endothelial repair (Gevaert et al., 2019). Restoring endothelial regenerative capacity could be a future target in HFpEF research.

### Cardiomyocytes

Brutsaert et al. discovered that besides vascular smooth muscle cells, cardiomyocytes are also influenced by the NO-sGC-cGMP pathway (Brutsaert et al., 1988). cGMP activates protein kinase G (PKG) in the cardiomyocyte, which acts as a brake on signaling pathways regarding cardiomyocyte stiffness and hypertrophy (Brutsaert, 2003). Stiffer, larger cardiomyocytes contribute to impaired active relaxation and passive stiffness. Through this pathway, endothelium-derived NO thus directly regulates cardiac diastolic function (Lim et al., 2015; Segers et al., 2018).

**Figure 3:**
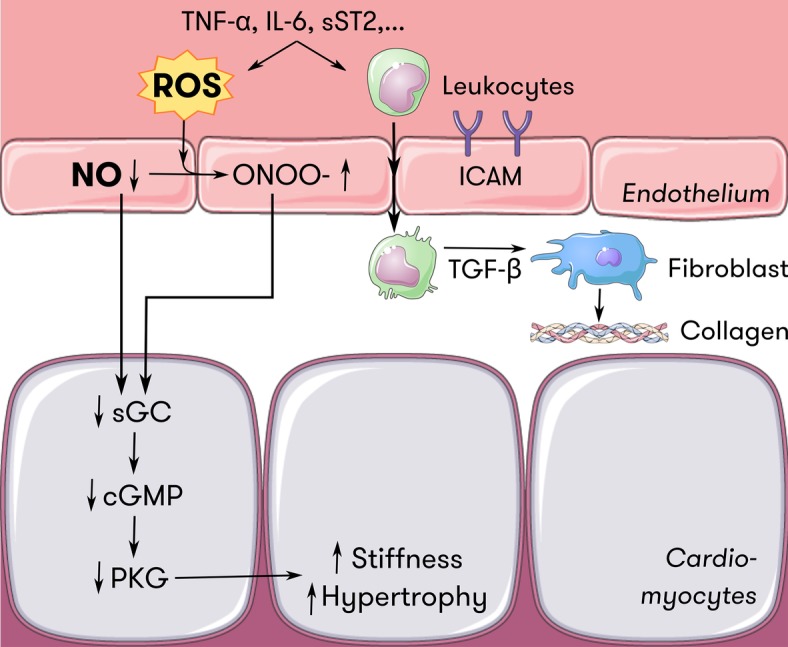
Endothelium-cardiomyocyte interaction in HFpEF. Inflammatory mediators such as TNF-α, IL-6, and sST2 induce oxidative stress at the level of the endothelium. ROS scavenge NO and induce ONOO^−^ formation, reducing the amount of NO available for diffusion to neighboring cells such as cardiomyocytes. Less NO diffusing to cardiomyocytes locally downregulates the sGC-cGMP-PKG pathway, which serves as a brake on cardiomyocyte hypertrophy and passive stiffness. Also, inflammatory mediators induce endothelial expression of adhesion molecules such as ICAM. Attracted leukocytes transmigrate and secrete TGF-ß, which stimulates collagen secretion in fibroblasts, thus contributing to ventricular stiffness. Adapted from Paulus and Tschöpe (2013) with permission. cGMP = cyclic guanosine monophosphate, HFpEF = heart failure with preserved ejection fraction, ICAM = intercellular adhesion molecule, IL-6 = interleukin-6, NO = nitric oxide, ONOO^−^ = peroxynitrite, PKG = protein kinase G, ROS = reactive oxygen species, sGC = soluble guanylyl cyclase, sST2 = soluble suppression of tumorigenicity 2, TGF-ß = transforming growth factor ß, TNF-α = tumor necrosis factor α.

In HFpEF, Paulus and Tschöpe hypothesized in 2013 that comorbidities such as aging, hypertension, diabetes, and obesity induce a systemic pro-inflammatory state (Paulus and Tschöpe, 2013). Circulating inflammatory cytokines increase oxidative stress at the level of the endothelium through activation of oxidative enzymes, reducing NO bioavailability. In the coronary microcirculation, this means that the NO-cGMP-PKG signaling pathway in adjacent cardiomyocytes is disrupted (van Heerebeek et al., 2012). Increased cardiomyocyte stiffness and cardiomyocyte hypertrophy follows, impairing diastolic function and starting the chain reaction of pathologic maladaptation leading to overt HFpEF (Leucker and Jones, 2014; Segers et al., 2018). This hypothesis is summarized in Figure 3.

The main molecular determinant of cardiomyocyte stiffness is the giant cytoskeletal protein titin. PKG is the most potent regulator of titin stiffness, by influencing its phosphorylation, isoform switching, and oxidative modifications (Hamdani et al., 2013b; Linke and Hamdani, 2014) Indeed, animal HFpEF models and cardiac biopsies of HFpEF patients exhibit an increased cardiomyocyte stiffness which is reversed by PKG administration (van Heerebeek et al., 2012; Hamdani et al., 2013a,b) and related to microvascular endothelial inflammation (Franssen et al., 2016b; Sorop et al., 2018). Of note, cardiomyocytes from HFpEF patients exhibit higher passive stiffness than HFrEF cardiomyocytes (van Heerebeek et al., 2006). Finally, cardiomyocyte stiffness is also influenced by diastolic calcium content and the associated regulatory proteins (Hamdani et al., 2013c; Røe et al., 2017). For a detailed description of these other mechanisms involved in passive cardiomyocyte stiffness, we refer the reader to other reviews (Linke and Hamdani, 2014; Franssen et al., 2016a).

Cardiomyocyte hypertrophy is an almost universal finding in animal and human HFpEF (van Heerebeek et al., 2006; Franssen et al., 2016b; Gevaert et al., 2017b). Besides the abovementioned NO-mediated mechanisms, cardiomyocyte hypertrophy is additionally induced by other molecular pathways. Hypertrophy can be induced directly by increased stretch on cardiomyocytes, through intrinsic mechanotransduction mechanisms (Lammerding et al., 2004). However, the relative contribution of mechanotransduction-induced hypertrophy is probably lower in HFpEF than HFrEF: cardiomyocyte diameters are larger in HFpEF, while wall stress is generally lower due to more concentric remodeling (van Heerebeek et al., 2006). Additionally, both angiotensin II and aldosterone cause cardiomyocyte hypertrophy independent of hypertension-associated wall stress increase, through upregulation of nicotinamide adenine dinucleotide phosphate oxidase (Li et al., 2002; Mihailidou, 2012). Thus, activation of the renin-angiotensin-aldosterone system, which is frequently seen in HFpEF patients, participates in maladaptive cardiac remodeling. For a detailed description of other pathways leading to cardiomyocyte hypertrophy, we refer the reader to other reviews (Heineke and Molkentin, 2006; Shimizu and Minamino, 2016).

### Fibroblasts

Passive LV stiffness in HFpEF is not only caused by cardiomyocyte stiffness, but also by changes in the composition and structure of the extracellular matrix, especially in fibrillar collagen. Cardiac biopsies from HFpEF patients show increased extracellular fibrosis (Mohammed et al., 2015; Zile et al., 2015). Likely, extracellular fibrosis is physiologically more important than cardiomyocyte stiffness in HFpEF, as LV end-diastolic pressure is only correlated to collagen-based stiffness, not to titin-based stiffness (Zile et al., 2015). Notably, microvascular changes (reduced capillary density) are strongly associated with the degree of extracellular fibrosis, again suggesting a link with microvascular endothelial dysfunction (Mohammed et al., 2015).

Of course, cardiac fibroblasts play a prominent role in the development of extracellular fibrosis. They reside in the extracellular matrix in a quiescent state, but are able to convert to collagen-secreting myofibroblasts after activation by inflammatory cytokines (Kendall and Feghali-Bostwick, 2014). Transforming growth factor ß is regarded as the most potent stimulus for myofibroblast differentiation. Again, endothelial dysfunction is considered a contributing factor. Circulating inflammatory cytokines induce expression of endothelial adhesion molecules such as vascular cell adhesion molecule and E-selectin (Westermann et al., 2011). Their expression promotes adherence and infiltration of monocytes, and polarizes macrophages infiltrated in cardiac tissue. Secretion of inflammatory mediators, including transforming growth factor ß, then induces myofibroblast differentiation and subsequent collagen secretion (Figure 3). Additionally, angiotensin II and aldosterone induce extracellular fibrosis through direct stimulation of collagen secretion by myofibroblasts, nicotinamide adenine dinucleotide phosphate oxidase activation, and suppression of matrix metalloproteinases (Murdoch et al., 2014; Jia et al., 2018).

### Experimental and Clinical Evidence for a Systemic Dysfunction of Endothelium-Cardiomyocyte-Fibroblast Interaction in Heart Failure With Preserved Ejection Fraction

The pro-inflammatory state in HFpEF is thought to be systemic. As such, inflammatory endothelial activation is probably not confined to the coronary circulation but present throughout the vasculature. A system-wide reduction in NO bioavailability could explain several pathophysiological findings in HFpEF, including reduced exercise-induced peripheral vasodilation, reduced vasoreactivity and vascular remodeling in the pulmonary arteries, reduced capillary density in the heart and skeletal muscle, and reduced renal blood flow (Figure 2; Shah et al., 2016; Gevaert et al., 2017a).

Our group and others have shown that endothelial inflammation and endothelial dysfunction are present in HFpEF animal models (Adams et al., 2015; Franssen et al., 2016b; Gevaert et al., 2017b). Interestingly, in aging mice developing HFpEF, the degree of endothelial dysfunction was comparable to that in “normally” aging mice. Endothelial inflammation, however, was present in aging mice but was incremental in aging mice with HFpEF (Gevaert et al., 2017b). This could point toward a higher relative importance of endothelial inflammation compared to endothelial dysfunction, when considering HFpEF pathophysiology.

In HFpEF patients, evidence is conflicting with regard to the presence of clinical endothelial dysfunction. This has been reviewed extensively recently (Gevaert et al., 2017a). In short, vascular function in large and medium-size vessels seems relatively preserved in HFpEF patients. However, almost all studies comparing microvascular endothelial function between HFpEF patients and matched healthy volunteers show a microvascular endothelial dysfunction in HFpEF (Borlaug et al., 2010; Akiyama et al., 2012; Lee et al., 2016; Gevaert et al., 2019).

### Knowledge Gaps

The hypothesis that links comorbidities, endothelial inflammation, and endothelial dysfunction is derived from rather circumstantial evidence. It is still unknown whether endothelial inflammation indeed leads to clinical endothelial dysfunction in HFpEF. Also, vascular dysfunction has been described in HFpEF patients in cross-sectional studies, but a causal relation is difficult to assess without longitudinal follow-up. Finally, the role of reduced numbers of endothelium-repairing cells in the development of endothelial dysfunction in HFpEF needs to be explored further.

## Risk Factors and Comorbidities

The noncardiac comorbidities associated with HFpEF lie at the root of the inflammatory endothelial activation (Franssen et al., 2016a). Besides female sex and increasing age, HFpEF is associated with obesity, diabetes mellitus, arterial hypertension, anemia, chronic obstructive pulmonary disease, and chronic kidney disease (Mentz et al., 2014). All these comorbidities can induce a systemic inflammatory state. In cross-sectional studies, potent inflammatory cytokines such as IL-6 and tumor necrosis factor alpha (TNF-α) are elevated in HFpEF patients and predict new onset of HFpEF in a community population (Kalogeropoulos et al., 2010; Collier et al., 2011). We zoom in on some of the most important risk factors and comorbidities below.

### Aging

The prevalence of HFpEF increases with age in both sexes (Dunlay et al., 2017). Besides an obvious role for an age-dependent increase in almost all other HFpEF risk factors, aging is thought to directly influence some of the pathophysiological mechanisms behind HFpEF.

LV stiffness increases progressively with age, and this increase is more prominent in women (Redfield et al., 2005). This leads to a higher prevalence of LV diastolic dysfunction with aging (Redfield et al., 2003). Underlying molecular mechanisms include increased transforming growth factor ß signaling and reduced expression of elastases leading to interstitial fibrosis, as well as mitochondrial oxidative stress, genomic instability, and epigenetic changes leading to altered calcium handling (Loffredo et al., 2014).

Aging is also linked with an increase in arterial stiffness and a reduction in endothelium-dependent vasodilation (Celermajer et al., 1994; Redfield et al., 2005; El Assar et al., 2012). Moreover, cellular endothelial repair declines with aging. Reduced numbers, migration, and proliferation of EPCs are seen in older individuals. This decline seems to relate to oxidative stress, as the number of EPCs inversely correlates with circulating levels of ROS (Ross et al., 2016). Also, numbers of circulating TAs are inversely related to age. Circulating EPCs and TAs are usually increased by exhaustive exercise; however, in healthy elderly, the exercise-induced increase in EPCs and TAs is attenuated (Ross et al., 2018).

We have shown in an aging mice model that, while aging leads to diastolic dysfunction without heart failure, adding a high-fat, high-salt diet leads to increases in vascular inflammation and signs of HFpEF (exercise intolerance, pulmonary edema, elevated LV filling pressures) (Gevaert et al., 2017b). Mice with HFpEF specifically showed an increase in cellular senescence in endothelial cells. Cellular senescence is a state of growth arrest linked to the aging process (Shakeri et al., 2018a). Premature senescence is thought to accelerate the development of cardiovascular diseases through continued secretion of inflammatory mediators (Shakeri et al., 2018b). These are secreted by senescent cells to signal their removal to the immune system, but they induce inflammation in the surrounding tissue and can accelerate senescence in neighboring cells, initiating a vicious circle of senescence and inflammation. In HFpEF, this “senescence-associated secretory phenotype” could be the missing link between aging and endothelial inflammation (Gevaert et al., 2017b).

### Female Sex

Both epidemiological studies and randomized trials consistently showed that most HFpEF patients are women (50–84%) (Lam et al., 2011). This sex bias can partly be attributed to the age distribution of the population at risk of HFpEF, as women have a higher life expectancy (Dunlay et al., 2017). In fact, when adjusting HFpEF incidence for sex-specific differences in age, obesity, blood pressure, drug treatment, and coronary artery disease, men are not at a significantly lower risk of HFpEF than women (Ho et al., 2016). The higher percentage of women in HFpEF populations can thus be explained by differences in demographic, anatomic, and risk factors. However, sex-specific analyses of large randomized trials do show a differential response to treatment. For example, North and South American women enrolled in the Aldosterone Antagonist Therapy for Adults with Heart Failure and Preserved Systolic Function (TOPCAT) trial had an improved prognosis when treated with the aldosterone antagonist spironolactone, while men did not (Merrill et al., 2019). Also, women show a better response to exercise training (Witvrouwen et al., 2019).

While we do not fully understand these divergent responses, perhaps the underlying molecular mechanisms leading to HFpEF differ according to sex. Endothelial dysfunction and arterial stiffness are less prominent in women (Beale et al., 2018), but on the other hand women are more prone to cardiac hypertrophy and fibrosis (Regitz-Zagrosek et al., 2010; Chen et al., 2015).

Also, the comorbidity profile differs between male and female HFpEF patients. Recent “big data” approaches to datasets of HFpEF risk factors have shown that different phenotypes can be identified, some of which exhibit a sex-specific dominance (Kao et al., 2015; Shah et al., 2015; Ahmad et al., 2018; Tromp et al., 2018). These phenotypes show a difference in treatment response and prognosis (Kao et al., 2015; Ahmad et al., 2018). Finally, regulation of genes and noncoding RNAs is highly sex-specific (Regitz-Zagrosek et al., 2010; Vidal-Gómez et al., 2018).

### Metabolic Syndrome

Obesity, arterial hypertension, and diabetes mellitus are common in HFpEF patients and often coexist (Mohammed et al., 2012). Arterial hypertension increases afterload on the LV, further increasing pro-hypertrophic signaling in cardiomyocytes and directly impairing ventricular-vascular coupling (Borlaug, 2014). Long-standing arterial hypertension also induces vascular remodeling leading to arterial stiffness (Redfield et al., 2005).

Obesity is a potent inductor of inflammatory signaling. Visceral adipose tissue is infiltrated by macrophages, which continuously secrete inflammatory cytokines (Berg and Scherer, 2005). Obese HFpEF patients also have an increased plasma volume, correlating with LV end-diastolic pressure (Obokata et al., 2017). Measures of body composition (body mass index) and more specifically of adiposity (fat mass index and leptin levels) also relate to exercise capacity in HFpEF (Carbone et al., 2016). This could be linked to adipose infiltration in skeletal muscles, which independently predicts peak oxygen uptake (peak VO_2_) (Haykowsky et al., 2014).

Diabetes mellitus can contribute to the development of HFpEF through several pathways. First, diabetes mellitus is associated with a systemic inflammatory state and increased oxidative stress, causing microvascular dysfunction and LV hypertrophy (Tabit et al., 2010). Second, diabetes accelerates atherosclerosis, leading to myocardial ischemia. Third, renal function is progressively impaired in diabetes, contributing to volume overload.

Arising from a systemic inflammatory state and increased oxidative stress, patients with hypertension, obesity, or diabetes also have impaired endothelial function and reduced EPC and TA levels (Brunner et al., 2005; Case et al., 2008; Tousoulis et al., 2008).

### Anemia and Iron Deficiency

Most data on anemia and iron deficiency in HFpEF are extrapolated from studies in HFrEF patients. Iron deficiency is the most frequent cause of anemia in HF patients, and both anemia and iron deficiency without anemia predict mortality (Okonko et al., 2011). Besides its well-known role in erythropoiesis, iron is also a key factor in mitochondrial metabolism, crucial for cells with a high energy consumption such as cardiac and skeletal myocytes. In HF, iron deficiency arises from nutritional defects, increased red cell destruction, hepatic congestion, inflammatory bone marrow dysfunction, and chronic kidney disease. Iron deficiency severely impacts functional status and exercise capacity (Jankowska et al., 2013). Regardless of the presence of anemia, intravenous correction of iron deficiency improves exercise performance, symptoms, and quality of life, and reduces hospitalizations in patients with HFrEF (Jankowska et al., 2016). Iron deficiency is thought to contribute to the development of HFpEF through increased oxidative stress and reduced mitochondrial function, but evidence is scarce (Paulus and Tschöpe, 2013; Hoes et al., 2018).

### Knowledge Gaps

While we know that abovementioned comorbidities contribute to endothelial dysfunction and diastolic dysfunction, the underlying mechanisms leading to these changes are unknown. Most animal models of HFpEF are young, male animals subjected to an acute event (surgery, salt overload) which does not accurately reflect HFpEF pathophysiology. Also, the relation between comorbidities and vascular dysfunction has never been examined specifically in HFpEF patients. Finally, an important barrier in identifying sex-specific underlying mechanisms of HFpEF is the predominance of male animals in experimental studies (Horgan et al., 2014).

## Genetic Regulation

Unlike HFrEF, in which several monogenic subtypes have been defined, little is known about potential genetic determinants of HFpEF (Tayal et al., 2017). Some genetic cardiomyopathies do exhibit a phenotype with preserved ejection fraction, for example hypertrophic cardiomyopathy and hereditary transthyretin amyloidosis. However, in most cases, it is difficult to discern genetic determinants of HFpEF from the influence of comorbidities: certain genetic factors may only be important in the presence or absence of a certain comorbidity. Thus, genetic determinants may differ between HFpEF phenotypes (Kao et al., 2017). An extensive review of gene reprogramming in HF falls beyond the scope of this review, we refer the readers to previous work (Dirkx et al., 2013; Deng, 2015). Recent advances have focused on the deregulation of noncoding RNA in HFpEF, which we summarize below.

### Role of the Noncoding Genome

Although about 75% of the human genome is transcribed, less than 2% is translated into proteins. However, the remaining “noncoding” transcripts do participate in regulation of biological processes, through interaction with coding RNA (Thum and Condorelli, 2015). MicroRNAs, short (20–25 base pairs) noncoding RNA molecules, are especially active as posttranscriptional regulators. They influence gene expression by binding to messenger RNA and causing its degradation or inhibiting its translation (Bartel, 2004). One microRNA can target hundreds of messenger RNA, and one messenger RNA can be targeted by several microRNAs, leading to an intricate network of posttranscriptional control. MicroRNAs are involved in all major biological processes and are implicated in several disease states, including cardiovascular disorders (Romaine et al., 2015; Schiattarella et al., 2018). Other noncoding RNA molecules (long noncoding RNAs, circular RNAs) are still poorly studied in HFpEF (Viereck and Thum, 2017).

#### MicroRNAs as Biomarkers

MicroRNAs can be secreted in the circulation, packed in exosomes and microparticles, or bound to lipoprotein complexes or RNA-binding proteins. These circulating microRNAs are stable in plasma and thus form attractive biomarkers (Valadi et al., 2007; Vickers et al., 2011). Cells release microRNAs in response to stimuli such as ischemia or cell death, and they can be taken up by target cells such as endothelial cells (Poller et al., 2018).

In HF, microRNAs have been investigated as possible biomarkers to aid in diagnosis. Several microRNAs provide benefit over traditional biomarkers to differentiate HFrEF from HFpEF (Watson et al., 2015; Wong et al., 2015). Also, several microRNAs are related to aerobic capacity or the response to exercise training (Sapp et al., 2017). MicroRNAs are released in the circulation after even short-term exercise, and training induces long-term changes in microRNA expression (Baggish et al., 2011; Uhlemann et al., 2014; Van Craenenbroeck et al., 2015).

#### MicroRNAs as Therapeutic Targets

As active participants in cellular cross talk, microRNAs are also attractive therapeutic targets. Inhibiting a microRNA or mimicking its activity potentially influences dozens of genes, which could lead to larger treatment effects compared to standard drugs (Poller et al., 2018). While microRNA-interfering therapy is still in its early developmental stage, several pilot studies have shown promising results in treating cardiovascular disease (Rupaimoole and Slack, 2017). For example, an inhibitor of microRNA-92a (which influences angiogenesis) improved blood flow after peripheral ischemia and enhanced recovery after myocardial infarction in mice (Bonauer et al., 2009). Inhibition of proapoptotic microRNA-34a or pro-fibrotic microRNA-21 improved LV function in mice with HF due to pressure overload (Thum et al., 2008; Bernardo et al., 2012). An increasing number of phase I and II clinical trials using microRNA therapy are being started (Rupaimoole and Slack, 2017). MicroRNA-based therapies for HFpEF are not yet under development, but some microRNAs have been identified as crucial regulators of pathophysiological processes underlying HFpEF, and are under investigation as therapeutic targets (Rech et al., 2018).

### Knowledge Gaps

As microRNA research is still emerging, little evidence exists, and many questions remain. Is there a mechanistic link between microRNAs and exercise intolerance in HFpEF? Or between microRNAs and vascular function? Is microRNA expression influenced by exercise training? What are the downstream targets of these deregulated microRNAs and can they become a novel therapeutic target? Can microRNAs be used as biomarkers to identify HFpEF phenotypes, or HFpEF patients with certain traits (e.g., responders to exercise training)?

## Effects of Exercise Training in Heart Failure With Preserved Ejection Fraction

While a drug therapy that improves prognosis or quality of life in HFpEF patients is still lacking, guidelines currently recommend exercise training as a therapy to improve aerobic capacity and quality of life in HFpEF patients (Ponikowski et al., 2016). This recommendation is based on the randomized multicenter Exercise in Diastolic Heart Failure (Ex-DHF) pilot trial and meta-analyses of several single-center trials that showed an improvement in peak VO_2_ and/or quality of life (Edelmann et al., 2011; Pandey et al., 2014; Fukuta et al., 2016).

How exercise training improves peak VO_2_ in HFpEF patients remains unclear. Following the Fick principle, i.e., VO_2_ = cardiac output × arteriovenous O_2_ difference, improvement in peak VO_2_ is caused by a cardiac factor (cardiac output), a noncardiac factor (peripheral O_2_ extraction), or both. In HFrEF patients, both cardiac and noncardiac factors are improved by exercise (Tucker et al., 2018). In HFpEF, several mechanisms have been proposed, investigated, and then refuted. Potential beneficial effects of exercise in HFpEF are displayed in Figure 1. Unraveling the benefits of exercise training in HFpEF is important, as its molecular determinants could hold the clue for novel pharmaceutical therapies, which will also benefit patients who are unable to perform exercise.

Improvement in diastolic function could be important as a “cardiac” mechanism. In middle-aged sedentary subjects, two years of exercise training was able to improve invasively measured ventricular stiffness (Howden et al., 2018). In HFpEF patients, the Ex-DHF pilot trial indeed showed an improvement in echocardiographic diastolic function with exercise training (Edelmann et al., 2011). However, several later studies could not confirm this finding (Smart et al., 2012; Fu et al., 2016; Kitzman et al., 2016).

As mentioned above, HFpEF patients also suffer subtle reductions in systolic function. Two studies showed no changes in peak cardiac output during exercise in HFpEF patients, but instead a significant change in arteriovenous O_2_ difference was observed (Haykowsky et al., 2012; Fu et al., 2016). The main sites for peripheral O_2_ extraction during exercise are the skeletal muscles. Skeletal muscle abnormalities are an often overlooked but clinically important feature of HFpEF patients: abnormal muscle mass, composition, capillary density, and oxidative metabolism have all been described (Kitzman et al., 2014). Although Fu et al. did show improved vastus lateralis muscle oxygenation in 30 HFpEF patients following a training program (Fu et al., 2016), no clinical study has specifically examined skeletal muscle abnormalities before and after exercise training in HFpEF. Animal studies suggest exercise improves muscle atrophy associated with HFpEF, although training did not affect muscle strength or fatigability (Bowen et al., 2017).

Skeletal muscle abnormalities can further contribute to exercise intolerance through overactivation of the autonomic nervous system (Piepoli and Crisafulli, 2014). Muscle atrophy in HF patients leads to enhanced sensitivity of muscle metaboceptors, which drive a feedback system called “ergoreflex” (Piepoli et al., 2006). The ergoreflex promotes hyperventilation, causing premature exercise discontinuation because of dyspnea. Ergoreflex activity is linked to the severity of HF and is increased in patients with decompensated HFrEF (Pardaens et al., 2014). In HFpEF, ergoreflex overactivity has been described in animal models, and was linked to the abnormal hemodynamic response to exercise in patients (Clifton et al., 2017; Roberto et al., 2017). Exercise training is able to reduce the overactivation of autonomic reflexes in HFrEF patients (Piepoli et al., 1996), but the effect in HFpEF patients has not been tested.

Peripheral O_2_ extraction also relies on appropriate distribution of blood to the peripheral tissues, and thus on normal endothelial function. By upregulating and phosphorylating endothelial NO synthase, reducing NO-scavenging free radicals, and increasing VEGF release, exercise training improves endothelial function (Haram et al., 2008; Adams et al., 2017). Clinically, endothelial function (FMD) can indeed be improved by exercise training in HFrEF patients (Pearson and Smart, 2017). However, in a single-center trial of 63 HFpEF patients, FMD was unchanged after 16 weeks of moderate aerobic exercise training despite an improved peak VO_2_ (Kitzman et al., 2013). Of note, in this study, diastolic function also remained unchanged after exercise, as well as arterial stiffness.

### Knowledge Gaps

Evidence regarding the cause of improvement in peak VO_2_ after exercise training in HFpEF is conflicting. Recent studies indicate that noncardiac improvements are more important, but whether this is due to improved peripheral vascular function or other factors (i.e., skeletal muscle metabolism) is still unknown. Moreover, the effects of exercise training on microvascular endothelial function and cellular endothelial repair in HFpEF are unknown. The recently completed multicenter Optimizing Exercise Training in Prevention and Treatment of Diastolic Heart Failure (OptimEx) and Ex-DHF 2 trials will soon shed more light on the benefits of exercise training in HFpEF.

## Summary

HFpEF is one of the largest unmet clinical needs in cardiology. It is a complex disorder with a central role for endothelium dysfunction induced by several comorbidities. Endothelial dysfunction leads to an intricate web of physiological abnormalities in the heart, blood vessels, and other organs. Several microRNAs have been identified as crucial regulators of pathophysiological processes underlying HFpEF, and could form interesting future diagnostic and therapeutic tools. Exercise training is currently the only available therapy to improve aerobic capacity and quality of life in HFpEF patients. Unraveling the underlying mechanisms responsible for this improvement could lead to new biomarkers and therapeutic targets for HFpEF.

## Author Contributions

AG and EV contributed to the conception and design of the paper. AG drafted the manuscript. JB, VS, and EV contributed to the critical revision of the manuscript. VS and EV obtained funding and supervised the work.

### Conflict of Interest Statement

The authors declare that the research was conducted in the absence of any commercial or financial relationships that could be construed as a potential conflict of interest.
